# A tale of two pathways

**DOI:** 10.7554/eLife.51990

**Published:** 2019-11-05

**Authors:** Jonathan A Oler, Julie L Fudge

**Affiliations:** 1 Department of PsychiatryUniversity of Wisconsin-MadisonMadisonUnited States; 2 Department of NeuroscienceUniversity of RochesterRochesterUnited States; 3 Department of PsychiatryUniversity of RochesterRochesterUnited States; 4 Del Monte Institute for NeuroscienceUniversity of RochesterRochesterUnited States

**Keywords:** connectivity, diffusion MRI, amygdala, prefrontal cortex, Human

## Abstract

A combination of invasive and non-invasive techniques has allowed researchers to take a closer look at the two major neural pathways that connect the amygdala and the prefrontal cortex.

**Related research article** Folloni D, Sallet J, Khrapitchev AA, Sibson NR, Verhagen L, Mars RB. 2019. Dichotomous organization of amygdala/temporal-prefrontal bundles in humans and monkeys. *eLife* **8**:e47175. doi: 10.7554/eLife.47175

Ever since the ill-fated explosion in 1848 that sent a tamping iron through the prefrontal cortex of railroad worker Phineas Gage, inexplicably changing his personality, scientists have wanted to understand how the different regions of the brain are connected (Harlow, 1999; Van Horn et al., 2012). The task is immense because the computational power of the human brain lies not only in its billions of neurons, but also in the connections between them. Short-term memory, decision-making, mood regulation, and other complex behaviors that depend on the prefrontal cortex (PFC) are all based on multiple, long-range connections between neurons.

The connectivity between the prefrontal cortex and the amygdala is believed to be critical for the regulation of emotion, and has been implicated in anxiety and mood disorders in humans (Kim et al., 2016; Tromp et al., 2012; Johansen-Berg et al., 2008; Riva-Posse et al., 2014). Importantly, invasive experiments on monkeys have revealed that the PFC and the amygdala are connected by two pathways: the amygdalofugal pathway and the uncinate fasciculus (Nauta, 1961; Price et al., 1987; Lehman et al., 2011).

Connectivity in the brain occurs through axons: projections of neurons that can extend over great distances to transmit information to target cells. Local activity in receiving neurons influences whether and how this information is passed on through output channels to other regions. Different techniques can be used to visualize axons extending between cells in the brain. Tract tracing studies in monkeys rely on the use of genetic or molecular markers, known as tracers, to follow the path of an axon. However, in order to visualize the tracers using a microscope, the brain has to be sliced and mounted on slides, making this a highly invasive technique. A neuroimaging technique called diffusion tensor imaging (DTI), also known as ‘tractography’, is non-invasive and can be used on humans.

Tractography produces images of the brain based on axon tracts – bundles of axons that travel in the same direction. This technique uses the fact that water diffuses in different directions in the brain depending on the structures it surrounds. This makes it possible to estimate the direction of the diffusion of water molecules along axons. Tractography works best when axons are myelinated and form large smooth tracts on the way to their destination. However, many pathways in the brain twist and turn, subdividing into smaller bundles, regrouping, and crossing one another. This makes tractography studies technically difficult due to problems with false negative and false positive findings.

Now, in eLife, Davide Folloni and co-workers at the University of Oxford and Radboud University Nijmegen (Jérôme Sallet, Alexandre Khrapitchev, Nicola Sibson, Lennart Verhagen and Rogier Mars) report on a study that combines data from tract tracing studies and DTI to more accurately visualize the connectivity between the amygdala and the PFC in monkeys and humans (Folloni et al., 2019).

Both the amygdalofugal pathway and the uncinate fasciculus curve and turn extensively, co-mingling with axonal pathways connecting other brain regions. The amygdalofugal pathway is particularly tricky, as it pierces an area of the forebrain known as the substantia innominata (meaning unnamed substance). As it goes through this region, the pathway splits into smaller axon bundles. Some of these axons leave the pathway here, innervating the cells within this area of the brain, while others continue on to the PFC. In this part of its route, the amygdalofugal pathway is crisscrossed by axonal tracts from other brain regions, which make it difficult to obtain clear images of the pathway using tractography.

Using DTI data from rhesus monkeys, Folloni et al. employed a probabilistic tractography method to estimate the most likely orientation of the axons as they cover the long distance from the amygdala to the PFC. Using known features of both the amygdalofugal pathway and the uncinate fasciculus, obtained from invasive tract tracing studies on macaques, they developed new methods for examining prefrontal connectivity in monkeys and humans. These tools helped to limit the problem of capturing crosstalk from intervening fiber systems, and allowed Folloni et al. to follow the predicted route of both pathways. They used known tract tracing results from macaques to validate their DTI approach, and then applied the validated DTI technique in humans. The results showed that the separation of the two pathways is conserved between humans and macaques.

In both species, they find that the amygdalofugal and uncinate pathways converge as they enter the lower PFC, before terminating in different regions of the frontal pole. The connectivity of both pathways was distinct from nearby control pathways. Folloni et al. then compared the connectivity ‘fingerprints’ of the two pathways in both species (i.e. the brain regions most and least targeted by the pathways), finding overall similarities but also subtle differences.

The findings from Folloni et al. underscore the critical importance of using nonhuman primates in psychiatric research due to the close anatomic match of evolutionarily expanded brains. Moreover, there is a critical need to validate and constrain DTI methodology using a ‘ground truth’ method. This new study shows the way forward by demonstrating the value of combining invasive and noninvasive data in cross-species analyses.

These new results can be useful for devising accurate biomarkers for psychiatric diseases and to understand how brain ‘wiring’ and myelination unfold with age in health and disease. Clinically, these methods may help identify pathways associated with specific symptom clusters found in different disorders, and help to provide individualized diagnosis. For example, some forms of depression may involve the amygdalofugal pathway, while other depressive illnesses, with different symptoms, might not. Localizing aberrant brain connectivity within a specific network linked to precise symptom profiles may help plan for emerging treatments such as deep brain stimulation or transcranial magnetic stimulation.
